# Bamboo, climate change and forest use: A critical combination for southwestern Amazonian forests?

**DOI:** 10.1007/s13280-019-01299-3

**Published:** 2019-12-03

**Authors:** Evandro Ferreira, Risto Kalliola, Kalle Ruokolainen

**Affiliations:** 1 Research Center of INPA in Acre, Estrada Dias Martins, 3868, Chácara Ipê, 69, Rio Branco, Acre 917-560 Brazil; 2 Department of Geography and Geology, University of Turku, 20014 Turku, Finland; 3 Department of Biology, University of Turku, 20014 Turku, Finland

**Keywords:** Deciduous forest, Fire, Forest management, *Guadua*, Rain forest

## Abstract

About 160 000 km^2^ of forests in the border zone between Brazil and Peru are dominated by semi-scandent bamboos (*Guadua* spp.). We argue that both predicted decreased precipitation during the dry season and widespread anthropogenic disturbances will significantly increase the distribution and biomass of bamboos in the area. Seasonal dryness favours the growth of evergreen bamboos in relation to trees that shed their leaves during the dry season. Disturbance can be beneficial for the bamboo because, as a clonal plant, it is often able to recover more rapidly than trees. It also withstands dry season better than many trees. The bamboo life cycle ends in a mass mortality event every 28 years, producing potential fuel for a forest fire. Presently, natural forest fires hardly exist in the area. However, in the projected future climate with more pronounced dry season and with increased fuel load after bamboo die-off events the forests may start to catch fire that has escaped from inhabited areas or even started naturally. Fires can kill trees, thus further increasing the fuel load of the forest. As a result, the landscape may start to convert to a savanna ecosystem.

## Introduction

Human activities affect climatic, edaphic and biological characteristics of practically all terrestrial ecosystems. Sometimes, the anthropogenic effects are deliberately planned and create immediate and obvious change in the ecosystem, like when an area of tall rain forest is converted to pasture by forest cutting and burning. The effect, however, is often unplanned, and the living conditions of people deteriorate. For example, decreased regional rainfall and increased flooding (Nobre et al. 1991; Guimberteau et al. 2017) are unwanted side effects of new agricultural lands in state-led agrarian reform in the Brazilian Amazon (Pacheco 2009).

Unplanned side effects are especially problematic if they are causal factors in a nonlinear change of an ecosystem. In such a change, the ecosystem remains practically stable until an unwanted state is triggered by relatively small transitions that exceed a threshold (Burkett et al. 2005). We argue here that the unplanned side effects of human activity, combined with regionally exceptional biological factors, are currently driving a large area of Amazonian lowland rain forest along the Brazil–Peru border towards a threshold where a forest ecosystem turns into a savanna ecosystem within the foreseeable future.

In lowland Amazonian rain forests, a sudden collapse and conversion to a drier forest or savanna has been suggested to occur before the end of this century under the pressure of global climate change enhanced by regional drying due to anthropogenic deforestation (Oyama and Nobre 2003; Cox et al. 2004; Malhi et al. 2008; Marengo et al. 2018). We believe that the forests of the border zone between Brazil and Peru are distinct from these Amazon-wide predictions because of a central role played by only two structurally distinct plant species in the otherwise species-rich tropical rain forest. Most of the forests in this area are dominated by two species of semi-scandent (up to 20 m tall) bamboos (*Guadua sarcocarpa* Londoño & Peterson and *G. weberbaueri* Pilger). Locally these woody grasses are known as *taboca* or *paca* (Silveira 2001; Carvalho et al. 2013; Dalagnol et al. 2018), hereafter collectively referred to as bamboo. Populations of the bamboo can grow as nearly pure stands, even several hectares in area, but they are more often intermingled with trees. The bamboo is a light-loving clonal plant that produces fast-growing scandent shoots capable of climbing over other plants. Additionally, it uses its subterranean rhizome to spread vegetatively to relatively open sites like natural treefall gaps or man-made forest clearings along roadsides or edges of pastures and agricultural land. Every 28 years, the bamboo flowers gregariously and dies as the seeds get ripe (Dalagnol et al. 2018).

Our standpoint is that, nowadays, the bamboo is strongly favoured by both the regionally drying climate, especially during the dry season, and the present human disturbance in the forests (Ferreira 2014). Human disturbance is most notable along the roads traversing the eastern and southeastern edges of the bamboo forest area in the provinces of Acre (Brazil) and Madre de Dios (Peru). Since the start of road construction projects in the 1960s, the population of the two provinces has grown by more than an order of magnitude and is now over 900 000. During this growth, large forest areas have been turned into pastoral landscapes (Velasco Gomez et al. 2015; Alarcón et al. 2016). Nonetheless, it seems likely that forest will remain as the main type of land cover. The deforestation rate has been relatively modest in recent years (INPE 2017; Global Forest Watch https://www.globalforestwatch.org/), and roughly 50% of the area with bamboo forests is protected as a national park, state forest, extractive reserve or indigenous territory (Acre 2006; Protected Planet https://www.protectedplanet.net/). But even if forest cover persists, it does not necessarily remain qualitatively similar as it was before. Extraction of both timber and non-timber forest products, even inside extractive reserves that form ca. 30% of the protected area, are increasing (Vadjunec et al. 2009). The removal of trees favours bamboo simply because the two compete with each other, but any activity that opens small clearings in the forest is likely to attract bamboo expansion.

Another consequence of increasing bamboo abundance is that the forest becomes more flammable, especially when there is dead bamboo biomass available over large areas as a result of the simultaneous bamboo die-off events. Currently, the climate is apparently wet enough to prevent major fires, but the drying climate in combination with increasing abundance of anthropogenic fires (Aragão and Shimabukuro 2010) can change the balance with potentially catastrophic consequences for the whole forest ecosystem.

Below, we describe in more detail how the bamboo forest area in southwestern Amazonia may be threatened by an irreversible ecological conversion to a more open habitat due to the simultaneous pressure from a variety of human-induced changes and the increasing abundance of the bamboo in the forest. We will approach this perspective by reviewing relevant literature on the ecology of the bamboo and the regional climate and vegetation and derive a detailed justification for a possible scenario for the bamboo forests of the region. The scenario can be summarised in three steps: (1) the abundance of bamboo in the forest is growing due to increasing forest disturbance created directly by human activities and indirectly via global and regional climate change that enhances tree mortality, (2) the increased bamboo biomass and more acute dry season droughts enhance the probability of canopy-devastating forest fires fueled by dead bamboos after a die-off event and (3) the destruction of the forest canopy and accompanying death of several canopy trees trigger the risk of a sudden switch from a forest to an open savanna ecosystem.

## Climate in the area of the bamboo forests

**Fig. 1:**
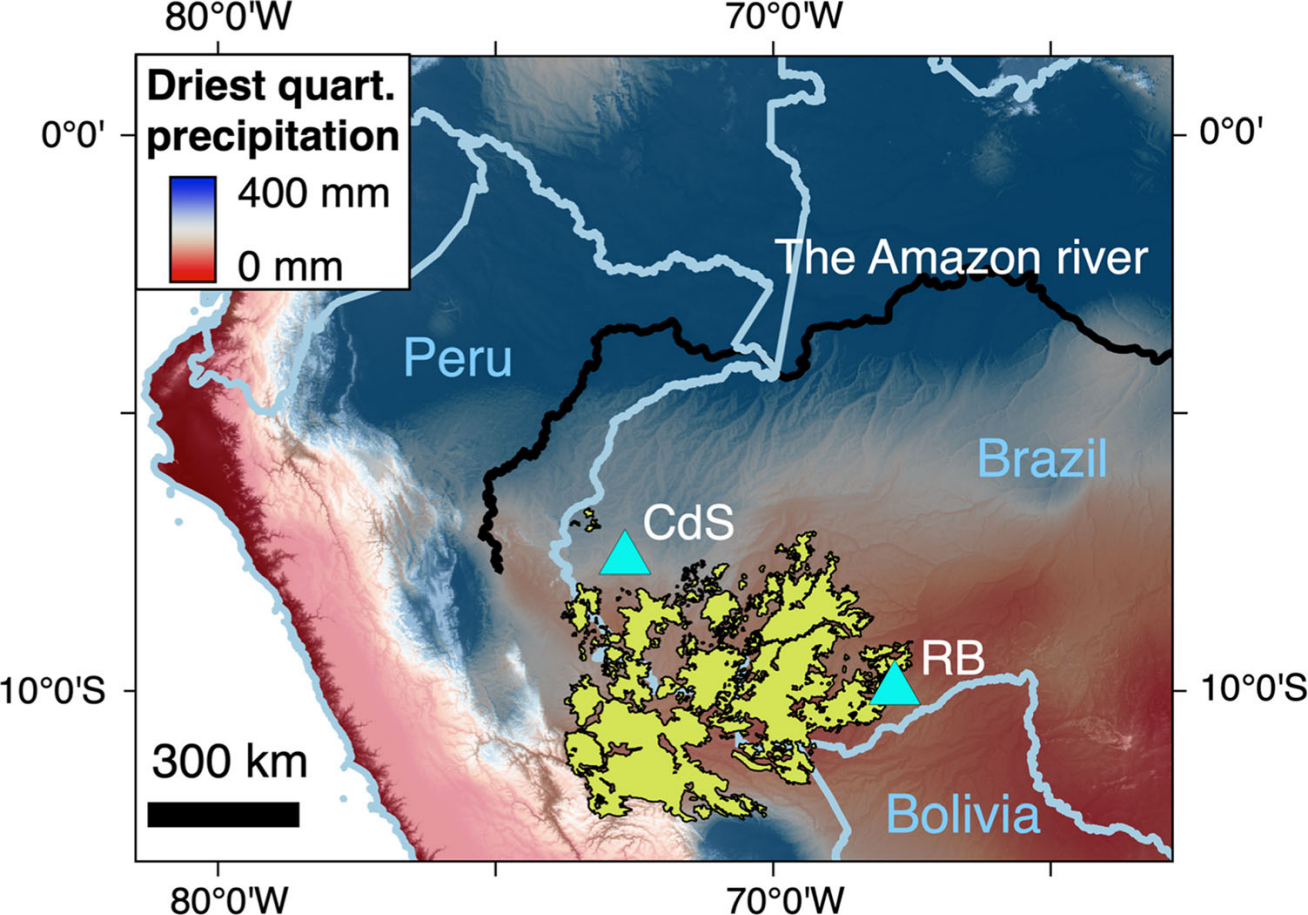
Average precipitation during the three driest consecutive months in western Amazonia. Precipitation data downloaded from the Chelsa site (Karger et al. 2017). The cities of Rio Branco (RB) and Cruzeiro do Sul (CdS) are marked with a triangle. The bamboo forests, as mapped by de Carvalho et al. (2013), are marked in yellow. The base map is the elevation model of the shuttle radar topography mission

**Fig. 2:**
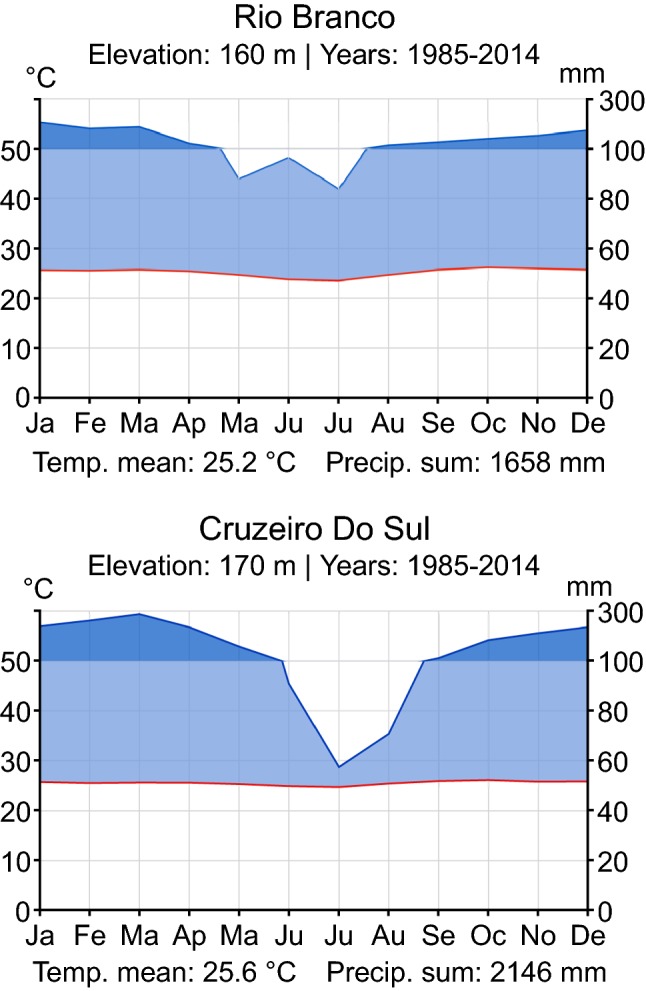
Walter climate diagrams for the cities of Rio Branco and Cruzeiro do Sul. The diagrams are modified from ClimateCharts.net

The climate in the southwestern part of the Amazon Basin is drier and more seasonal than in equatorial Amazonia (Figs. 1, 2). The temperature is rather constant during the year (24–26°C) and regional differences are small. The annual average precipitation is around 2000 mm, showing a gradient from the drier southeast to a wetter northwest (Espinoza Villar et al. 2009; Santos et al. 2015).

Historical data on annual precipitation from the area show hardly any evidence of change over the last 90 years (Marengo 2004; Almeida et al. 2016). Nevertheless, it is justified to say that a trend of increasing dryness has been observed as the amount of water available for plants has decreased due to rising temperatures. The result is an increased reference evapotranspiration over the last 30 years (Almeida et al. 2016; da Silva et al. 2016). Furthermore, it has been observed that dry seasons have become longer over the whole of southern Amazonia (Fu et al. 2013).

Based on climate models, it is not clear whether the observed tendency towards drying will continue in southwestern Amazonia (Good et al. 2013). The area is situated quite exactly in a transition zone between southeastern Amazonia, which is predicted to become drier, and the presumably wetter northwestern Amazonia (Malhi et al. 2008; Marengo et al. 2012; Ramos da Silva and Haas 2016; Souza et al. 2016). Accordingly, a rather slight deviation from the predicted climate trajectory can turn the outcome to either a wetter or drier direction.

However, there are reasons to believe that the drier option for the future climate in southwestern Amazonia is more likely than expected when looking only at the climate models. Barkhordarian et al. (2017) found that all the global and regional models failed to reproduce the observed warming during the austral spring in northern South America in 1983–2012. They were able to show that this discrepancy can be explained, in large part, by the effect of anthropogenically produced aerosols. An additional factor not included in the climate models, which pushes the future regional climate in a drier direction, is deforestation. The replacement of trees by pastures, fields and secondary vegetation is likely to support local or even regional drying of the climate (Laurance and Williamson 2001; Aragão et al. 2008; Cochrane and Laurance 2008). At any rate, increased human presence and disturbance means that anthropogenic fires are going to be more common in southwestern Amazonia, and some are likely escaping to forests (Nepstad et al. 2008).

An aspect upon which most future climate projections agree for southwestern Amazonia is that variability both in precipitation and temperature is going to significantly increase, even from the presently rather high levels affected by the El Niño–Southern Oscillation or ENSO (Marengo and Espinoza 2016; Fonseca et al. 2017; Bathiany et al. 2018). Consequently, events of both extreme dryness and exceptional flooding are going to become more frequent. This will increase the risk for forest fires, even if the average climate remains the same or even turns somewhat moister.

## Bamboo forest ecology and human intervention

The western Amazonian bamboo forests can be mapped (Fig. 1) at least roughly using satellite images because the foliage of the bamboo is able to reach the upper canopy layer, and its reflection of light is distinct from that of trees. A recent estimate is that the bamboo forests cover over 160 000 km^2^ of southwestern Amazonia (de Carvalho et al. 2013), but this might be an underestimate (Dalagnol et al. 2018). The bamboo forests are not uniform but are variable according to the density of the bamboo and accompanying arboreal species (Silman et al. 2003; Acre 2006; Castro et al. 2013; Cornejo Valverde 2016; Yavit 2017).

According to our study of old satellite images in public archives, bamboo forests have also occurred in many areas that are now deforested, particularly in eastern Acre. From a longer temporal perspective, it appears evident that the bamboo and bamboo-dominated forests have been present in the area for a considerable time. Direct evidence comes from a macrofossil of ca. 45 000 year BP (Olivier et al. 2009) and from archaeological excavations where bamboo phytoliths were discovered from several thousand-year-old anthropogenic deposits (McMichael et al. 2014; McMichael et al. 2015). There are also animals, especially birds and insects, that are specialised for living in bamboo thickets (Kratter 1997; Jacobs et al. 2012), implying that the habitat has existed for long enough to allow for the evolution of such habitat specialists.

The bamboo-dominant landscape is dynamic over time because the bamboos are gregariously semelparous, i.e. all the individuals of a population flower simultaneously and only once per lifetime. After flowering and shedding the fruits, the whole bamboo population dies. The bamboo life cycle takes 28 years, and the geographical area of a synchronously flowering population typically covers some hundreds of square kilometres but can extend to even some thousands of square kilometres (de Carvalho et al. 2013; Dalagnol et al. 2018).

The growth phase of the bamboo life cycle is a battleground of bamboos and trees. After the fruiting, the dying bamboo culms shed their leaves and the ground level receives much more light than was available before. At that moment, rapidly germinating bamboo seeds are present and the emerging seedlings will enjoy the benefit of increased light. We have seen situations in which abundant bamboo seedlings practically take over a site. But apparently the bamboo does not always come out as the winner in the competition because otherwise it would be difficult to understand how the bamboo density can vary from strong dominance to complete absence within a short distance in a uniform-looking terrain.

It would be crucial to know how bamboo abundance can vary in the long term within a single site and what might be the local factors that eventually lead to the dominance of either the bamboo or trees. As trees are economically far more important than the bamboo, the research is biased towards documenting cases in which trees are losing the terrain and being harmed by the bamboo, whereas documentation of the opposite (Socolar et al. 2013) remains anecdotal. At any rate, the competition by bamboo generally reduces the vigour, diversity and total biomass of trees by arresting forest succession (Griscom and Ashton 2006). Tree seedlings and saplings easily fail to grow up as bamboo culms are constantly growing and collapsing over them (Griscom and Ashton 2006). In addition to the documented physical arboreal damage that the bamboo is causing, we believe that the bamboo gains a competitive edge over trees, especially during the dry season, as it is likely able to store water in its extensive rhizomes and/or may utilise the water that accumulates in its internodes. Whatever the physiological mechanism, the advantage exists because the bamboo is evergreen, whereas, apparently, a big proportion of trees with which it is competing are deciduous. When the trees are leafless and therefore not photosynthesising, the bamboo is found to grow both above and below ground, albeit more slowly than during the rainy season (Silveira 2001).

We can provide some circumstantial evidence to support our suggestion that the bamboo attains dominance in the subcanopy only when the dry season is strong enough to make the forest semi-deciduous. Firstly, a relatively low level of precipitation, especially during the dry season, correlates with the distribution of bamboo forests. This is because the area mapped as bamboo forest (Fig. 1) has, on average, only 58% of the amount of precipitation that falls on the 200 km–wide buffer around it during the driest quarter of the year, whereas during the wettest quarter, that amount is 84% (116.3 vs. 201.0 kg/m^2^ of rain during the driest quarter and 755.5 vs. 897.3 kg/m^2^ during the wettest quarter; precipitation data from CHELSA and only from altitudes below 700 m above sea level for both areas; Karger et al. 2017).

**Fig. 3:**
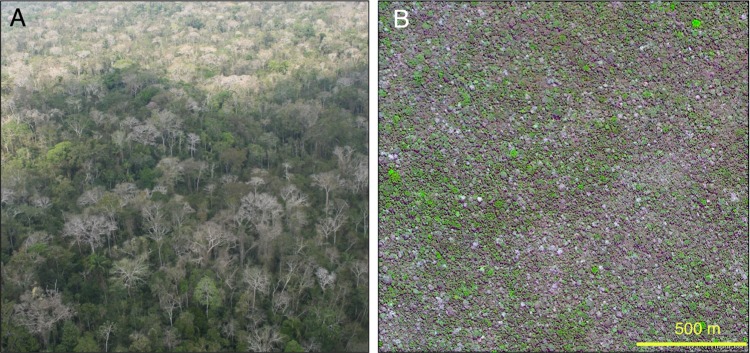
A. Aerial view from the Chandless State Park in central Acre showing abundant deciduous trees during the dry season (photo: E. Ferreira 2008). B. Green and leafless tree canopies in southern Acre (Digital Globe image, 28.9.2012, 9° 58′ S, 70° 37′ W)

Secondly, the tree canopy in the bamboo forest area should probably be described as semi-deciduous (Ferreira 2014) instead of evergreen, as is done in standard classifications (Olson et al. 2001; IBGE 2004). One of us (EF) had the opportunity to fly several times over Chandless National Park in central Acre during the dry season (June–August) between 2005 and 2008, when the management plan of the park was produced. During the flights, it was possible to observe that perhaps even 50% of canopy trees were leafless (Fig. 3a). We also examined Google Earth satellite imagery to find evidence about the possible semi-deciduousness of the forests. Google Earth shows some swaths of high-resolution (0.5 m) Digital Globe products during the dry season. In those images, it is possible to see that leafless trees can indeed be quite common, especially towards the end of the dry season (Fig. 3b). In contrast, hardly any tree canopies are leafless in the same products representing practically aseasonal forests north of the bamboo forest area. Many southwestern Amazonian canopy tree species are known to shed their leaves during the dry season (Pitman et al. 2001; Freeman et al. 2002; da Cunha et al. 2016), but we are not aware of any quantification of deciduousness among canopy tree individuals.

After a die-off event, dead bamboo biomass abounds and provides possible fuel for forest fires. It has even been suggested (Keeley and Bond 1999) that regular fire is the main disturbance factor favouring the existence of the bamboo. The relatively scarce occurrence of charcoal in forest soils of the region (McMichael et al. 2013), however, suggests that wild fires hardly have been common in the moderately distant past. Fire does not seem to be a relevant element of the forests in the present climatic conditions, either. Studies based on both MODIS (Shimabukuro et al. 2009; Dalagnol et al. 2018) and Landsat (da Silva et al. 2018) data show that, over the 34 years between 1984 and 2017, there have been forest fires in Acre in less than 5% of the forest area (Shimabukuro et al. 2009; da Silva et al. 2018). Most importantly, none of these fires occurred in uninhabited areas, but all were found either in the Chico Mendes Extractive Reserve in southeastern Acre or elsewhere in the immediate vicinity to agricultural areas. However, the exceptionally dry years 2005 and 2010 accounted for 90% of the fires (da Silva et al. 2018), giving a taste of fire frequency in the possibly drier future climate.

The bamboo seems to recover well from a forest fire because the underground rhizome rather easily survives the fire. In fact, forest fires may benefit the bamboo, at least in the short run. The stored resources of the rhizome allow for vigorous regrowth, which enables them to win over other plants that are competing for space in the burned area (Smith and Nelson 2011; Barlow et al. 2012; Numata et al. 2017). We have witnessed this mechanism in recently burned agricultural fields where bamboo regrowth competes with crop plants. On the other hand, we believe that for the long-term persistence of the bamboo it is actually crucial that fires are quite rare. The die-off phase of the bamboo life cycle produces an abundance of easily drying fuel, and if that catches fire, the seeds and seedlings of the next generation would hardly have a chance to survive the flames. Seeds of the bamboo cannot escape the fire through dormancy, as they germinate almost immediately after having shed. Young seedlings, in turn, are vulnerable as they lack rhizomes that would allow them to survive the flames.

**Fig. 4:**
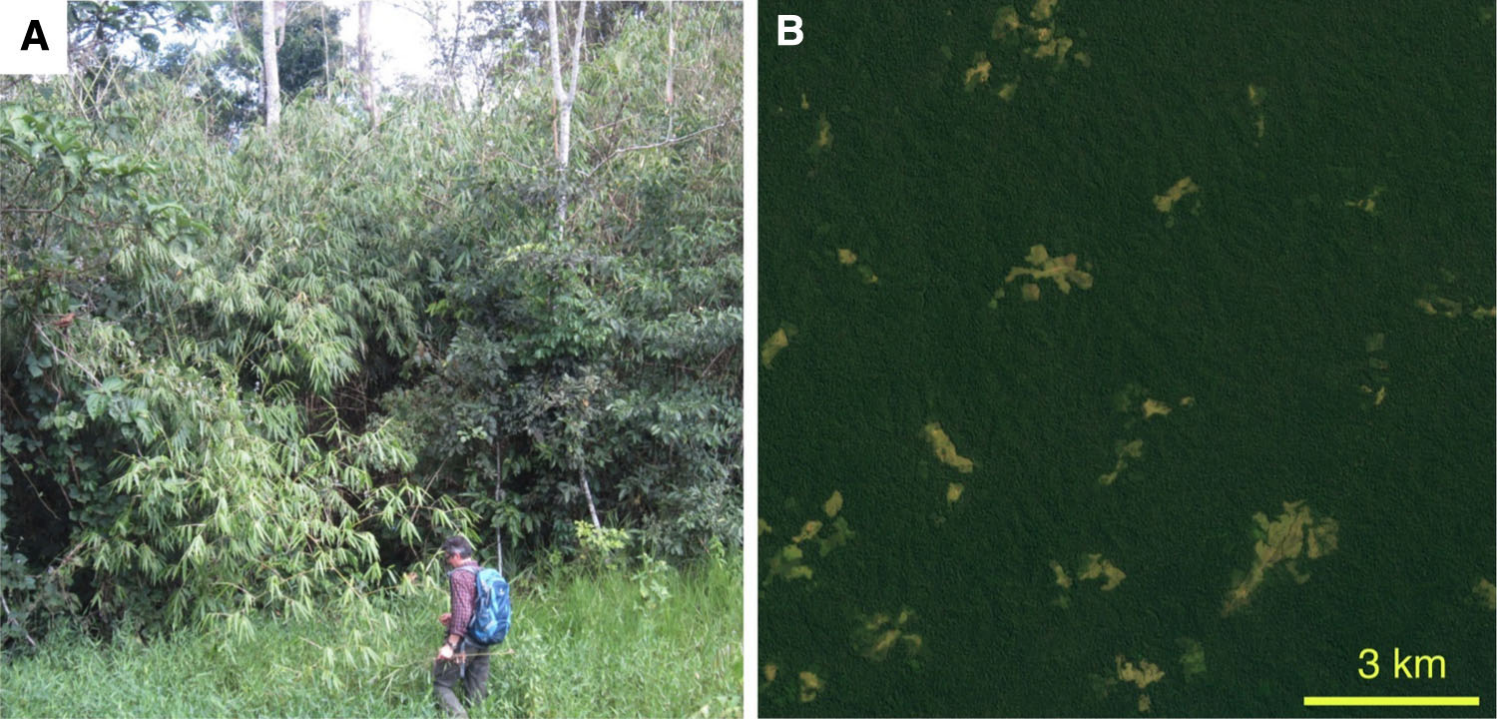
**a** Bamboo stand occupying a roadside in southeastern Acre (10° 4′ S, 67° 36′ W; photo: R. Kalliola 2017). **b** Forest dotted by household-sized forest clearings in the Chico Mendes Extractive Reserve (10° 38′ S, 69° 17′ W; image from Google Earth)

The bamboo needs light, and therefore, natural forest openings attract nearby bamboo clones into their margins. Also, the edges of forest roads, trails and the clearings made for individual households or hamlets often have bamboos around (Fig. 4a). Small forest clearings scattered all around the landscape are particularly characteristic in the Chico Mendes Extractive Reserve in Acre (Fig. 4b). Bamboos also benefit from the widespread tree felling sites in many parts of southwestern Amazonia. Analysis based on multi-temporal Landsat Enhanced Thematic Mapper Plus (ETM +) satellite data showed an average impact area of Acre selective logging as 76 km^2^ year^−1^ during 1999–2002 (Asner et al. 2005). The actual rate is probably much higher since small-scale tree extraction is hard to detect from this imagery (Milodowski et al. 2017). One should realise, though, that not every forest opening will be conquered by the bamboo. It happens only when bamboo is already present in the cleared area or its immediate vicinity. Seed production is a rare event, and seed bank does not exist, so the bamboo evidently cannot easily colonise areas where it is not already present.

## The possibility of passing a threshold between forest and savanna

**Fig. 5:**
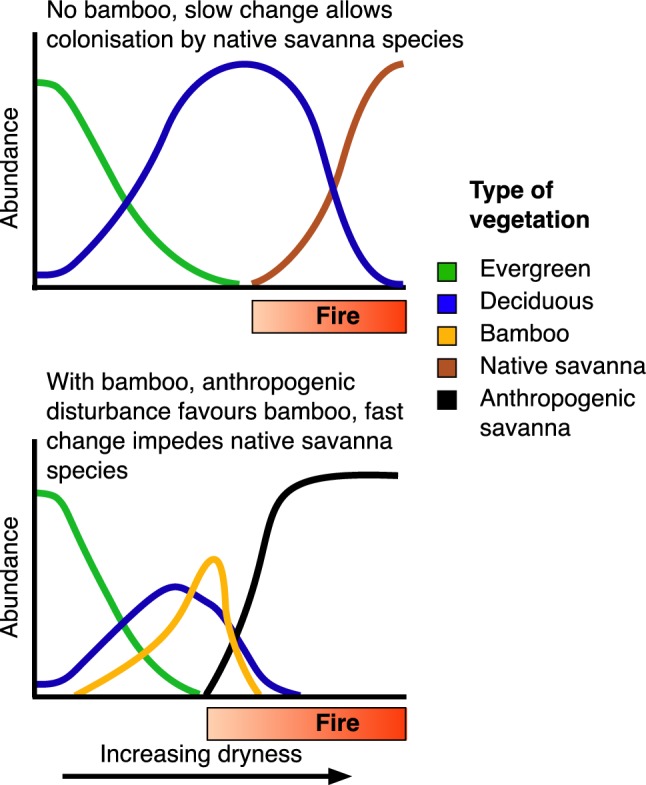
A possible scenario of the future of the bamboo forest area in southwestern Amazonia. The upper diagram depicts tropical vegetation along a natural gradient of increasing dryness without the presence of bamboo. The evergreen forest gives way to deciduous vegetation that turns to savanna by the effect of fire. The lower diagram illustrates the same gradient with the presence of mast flowering semelparous bamboo under a rapid change of climate and extensive anthropogenic disturbance. The bamboo competes especially with deciduous trees. Bamboo die-off events increase the flammability of the vegetation, allowing fire to affect vegetation in wetter climatic conditions than would happen without the bamboo. Fires consuming dead bamboo are so strong that they kill native deciduous trees. Dead trees provide fuel for further fires. The vegetation turns to savanna, consisting of a mixture of introduced weedy species and native savanna species that manage to disperse to the area from existing savannas that lie some two hundred kilometres away in Bolivia

The review above suggests that several concurrent conditions in southwestern Amazonia synergistically favour bamboo growth and at least local vegetative expansion. The increase of bamboo due to anthropogenic disturbance and drying climate may lead to severe forest fires that can result in large-scale, permanent forest degradation (Fig. 5). The foremost driver for this perspective is the changing climate, particularly the dry season conditions becoming increasingly harsh.

There are no precise forecasts specifically for southwestern Amazonia about possible future vegetation, but regional scenarios for the whole of tropical South America and Amazonia do exist (Salazar et al. 2007; de Lyra et al. 2016). In these studies, the most pessimistic climate change predictions suggest that southwestern Amazonian forests would practically disappear and be converted to savanna by the end of this century. The less dramatic predictions identify variable proportions of evergreen forest, deciduous forest, savanna and grassland. The possible effects of deforestation were not taken into account in these studies, but more recent models (Llopart et al. 2018) suggest that deforestation will further decrease precipitation, especially in western Amazonia. In these scenarios, we believe that the bamboo is an important neglected factor. The bamboo benefits both from the drying climate and from human disturbance. Recent ENSO years with severe drought periods have already hit this area strongly (Lewis et al. 2011). The 2005 dry season helped researchers anticipate possible future events, as it showed the selective mortality of many forest trees (Phillips et al. 2009). If deciduousness increases, it may favour bamboos, thereby decreasing the volume of commercial timber species (Rockwell et al. 2014). Abundant bamboo may also negatively affect the public opinion about the value of the forest simply because the spiny bamboo thickets are neither easy nor pleasant to enter.

It seems reasonable to assume that a die-off patch of the bamboo is easily flammable after a dry spell dries it out. Indeed, it has been observed that, especially in the dry years, recently dead bamboo forests catch fire more easily than actively growing bamboo forests do (Dalagnol et al. 2018). Also, a study on *Melocanna baccifera* bamboo forests in Myanmar showed that, after a die-off event, the burned areas in bamboo forests more or less doubled (Fava and Colombo 2017). Adding human disturbance to increased flammability easily leads to wildfires as seen in the more southern parts of Amazonia (Cochrane and Laurance 2008; Devisscher et al. 2016). Since an average patch of a bamboo die-off is very large, the moment it catches fire, a sizable chunk of thousands of square kilometres of forest can burn in a single blow. Furthermore, the amount of easily flammable biomass provided by dead bamboo per area may be high enough to create a fire that reaches the canopy and kills even big trees. Such stand-devastating fires fueled by another *Guadua* bamboo are known from the disturbed mesic *Nothofagus* forests in northern Argentina (Veblen et al. 1992).

If a whole patch of bamboo die-off burns, it is not at all evident that the area will be regrown by the same species of the semi-deciduous forest. Firstly, a strong fire will kill several trees, and their dead biomass will provide fuel for fire during the subsequent dry seasons. Secondly, bamboo patches of a synchronous reproductive rhythm are typically large, reaching hundreds or even thousands of square kilometres (de Carvalho et al. 2013; Dalagnol et al. 2018), and therefore, destruction of trees over such an area can have a similar drying effect on the local climate as deforestation has. Thirdly, the decline in canopy cover and live biomass and the difficulty of poorly dispersing plants to send propagules far from the edge of intact forest favour the immigration of opportunistic pioneer species representing early successional forest trees and both exotic and native invasive shrubs, herbs and grasses (Veldman and Putz 2011). Therefore, the burned bamboo forest may turn into a shrub savanna in which the species composition does not correspond to any natural savanna. In Bolivia, such derived savannas were found to emerge especially in sites with relatively nutrient-rich soils (Veldman and Putz 2011), and relative fertility also characterises the soils of bamboo forests (de Carvalho et al. 2013). The derived savannas of Bolivia had a higher fuel load than natural ones, and this feature may contribute to regular fires that hamper or exclude tree regeneration (Brooks et al. 2004; Brando et al. 2014).

We recognise that the above-described shift from a semi-deciduous forest to a regularly burning open grassland system seems rather speculative because of the uncertainties about the magnitude (and even the direction) of future climatic change in the area (Good et al. 2013). However, it is clear that the current climate is quite close to the minimum level of moisture needed to maintain a semi-deciduous forest. Also, the bamboo die-off events are producing large amounts of flammable biomass over extensive areas at a certain time interval. Furthermore, it is known that on the climatic forest–savanna ecotone, the forest and savanna can be regarded as two alternative stable states, chosen according to fire frequency (Hirota et al. 2010; Hirota et al. 2011; Oliveras and Malhi 2016).

There is an active discussion as to whether global climate change and regional deforestation make Amazonia so much drier that large areas of lowland rain forest can reach a tipping point after which the ecosystem suddenly turns to savanna (Boulton et al. 2017; da Silva et al. 2018; Marengo et al. 2018; Ambrizzi et al. 2019; Goodman et al. 2019; Rangel Pinagé et al. 2019). We share this scenario but argue that, in the large bamboo-dominated forest area in southwestern Amazonia, the tipping point is going to be reached with a smaller increase in dryness. Our hypothesis is that the vegetative bamboo phase may reduce tree biomass locally and that the regular bamboo die-off events provide a fuel load that makes it easier for forest fires to reach an intensity that is sufficient to kill the canopy trees. This hypothesis can be tested experimentally by burning dried-up patches of forest with recently dead bamboo and without bamboo. It would also be critical to investigate whether bamboo seeds and seedlings are indeed susceptible to fire because, if not, then the likely result is only an increased bamboo dominance in the forest and not a switch to a savanna ecosystem.

## Potential to reduce the risk

Obviously, all measures that slow down either global or regional anthropogenically induced climate change will alleviate the risk of reaching a tipping point that turns southwestern Amazonian bamboo-dominated forests to savanna. Additionally, this risk can become lower by taking actions that lower the abundance of the bamboo in the forest. From a forestry point of view, the bamboo is a nuisance species (Griscom and Ashton 2006), and there has been some interest in finding management practices that would diminish its negative effect. One practical suggestion is to cut timber trees at a short and low-intensity exploitation cycle, alternating the exploited species (d’Oliveira et al. 2013). Each intervention supposedly pulls down bamboo thickets, and the remaining trees may benefit from this temporal decrease in bamboo. This suggestion, however, seems somewhat controversial, as the long-term net effect of forest disturbance by timber extraction may also turn out to benefit the bamboo. It would be better to coincide the timber exploitation with the events of bamboo die-off and fruit production or early development of seedlings as often as practically possible (Rockwell et al. 2014). One should also try to control anthropogenic fires as much as possible and ensure the reforestation of abandoned anthropogenic forest clearings by trees.

The threat of a tipping point that we see for these western Amazonian rain forests is peculiar because it is closely attached to the biological characteristics of just two closely related species. Furthermore, both of the species are native to the exceptionally species-rich environment, and the two can be seen as a key resource for several specialised animal species. Therefore, even if reducing the abundance of bamboo is beneficial for the preservation of the forest, the bamboo is such an integral part of the ecosystem that any measures to prevent it should also be carefully weighed against possible drawbacks in species conservation. It is quite clear that more studies are needed on both the basic biology of the bamboo and on the regional details of climate change in order to make wise management and conservation decisions about these forests.
